# Bells and Whistles on Fertilizers: Molecular Hands to Hang Nanoporous Foliar Fertilizer Reservoirs

**DOI:** 10.1021/acsomega.3c09895

**Published:** 2024-06-05

**Authors:** Kamaljit Kaur, Mahima Chandel, Poonam Sagar, Bandana Kumari Sahu, Ritu Ladhi, Parameswaran Rajamanickam, Pooja Aich, Madhu Khatri, Selvaraju Kanagarajan, Nitin Kumar Singhal, Monika Singh, Vijaya Kumar Shanmugam

**Affiliations:** † University Institute of Engineering and Technology, Panjab University, Chandigarh 160014, India; ‡ Institute of Nano Science and Technology, Sector- 81, S.A.S. Nagar, Mohali 140306, Punjab, India; § Food and Nutritional Biotechnology, National Agri-Food Biotechnology Institute, Mohali 140308, Punjab, India; ∥ Department of Plant Breeding, Swedish University of Agricultural Sciences, 234 22 Lomma, Sweden

## Abstract

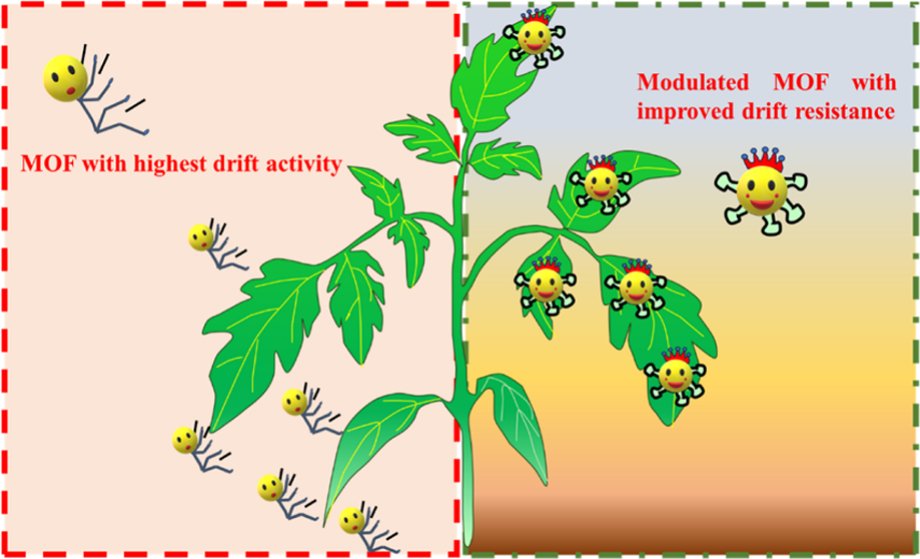

Porous materials are highly explored platforms for fertilizer delivery. Among porous materials, metal–organic frameworks (MOFs) are an important class of coordination polymers in which metal ions and organic electron donors as linkers are assembled to form crystalline structures with stable nanoporosity. Selected amino acids were inherently found to have the capacity to hold the leaf cuticle. Hence, MOF synthesis was attempted in the presence of amino acids, which can act as surface terminators and can assist as hands to hold to the leaf for a controlled nutrient supply. By serendipity, the amino acids were found to act as modulators, resulting in well-stabilized porous MOF structures with iron metal nodes, which are often noted to be unstable. Thus, the composite, i.e., (MOF@aa) MOF modulated with amino acids, has efficient nutrient-feeding ability through the foliar route when compared to the control.

## Introduction

Iron deficiency in humans is alarming, which is because of the deficiency in primary human nutrition sources, viz., agricultural products. Different ways to enrich nutrients in food include the agronomic route, breeding route, and finally direct fortification with salts after harvesting. In spite of iron being so abundant in the soil, its deficiency is often noticed in crops due to its presence in phyto-unavailable forms, i.e., oxy and hydroxy forms. Even externally applied iron fertilizer salts quickly turn into phyto-unavailable forms because of the carbonate ions in the soil, especially in calcareous regions, which are rapidly expanding. Materials can be remarkably tuned to extended fertilizer release and bioactivity through sustainable release diffusion control and smart engineering.^1−5^ Thus, organic–metal hybrid porous materials have been tested and used as carriers for agrochemicals such as fertilizers.^6^ Engineered porous silica is known to be compatible, and its transport in the plant has also been documented.^7^ Engineered porous silica matrices with biopolymer encapsulation have been shown to selectively open for the root uptake signal and close to the soil carbonate ions for efficient iron supplementation.^8,9^

Metal–organic frameworks (MOFs) are another class of porous materials with more surface area in comparison to that of porous silica. MOFs are coordination polymers having metal ions as nodes and organic electron donors as linkers with a flexible architecture.^10^ In its quick phase of growth, various molecules have been tested as modulators to stabilize the structure; in this line, biomolecules, viz., amino acids, exhibit a mosaic of structures.^11^ MOF applications in catalysis, energy storage, water filtration, and gas adsorption have been explored.^12−16^ Also, transition-metal-based MOFs have also been explored for ion-sensing and photocatalytic water splitting.^17−20^ Further, in biology, the porous architecture has been used for sustained drug delivery.

Among agricultural applications, the value of MOFs in vegetable oil esterification, saponin conversion, and cellulose modification has gained appreciation.^21−23^ The compatibility of MOFs with different substitute metals, such as Cu/Al and Ti, helps tune their properties for better pesticide adsorption and photocatalytic degradation, respectively.^24−26^ On the other hand, MOFs with an Fe node were shown to protect pesticides from photodegradation and extend their efficiency.^27^ Interestingly, MOFs have been tested as fertilizer carriers for nitrogen and phosphorus application to plants, which conveyed excellent nutrient-feeding efficiency to the roots.^28^

However, Fe applied to plants undergoes undesired conversion in the complex soil matrix; hence, our previous design using mesoporous silica was found to be more suitable for hydroponic applications only.^8^ Foliar application is another wise way to feed nutrients as the chances of undesired conversion are limited. However, unfortunately, it is prone to drift, and the direct nutrient spots cause leaf burn. MOFs can act as reservoirs for slow release so that continuous nutrient support can be achieved. MOFs are susceptible to metal node replacement and reversible exchange,^29^ which may collapse the porous structure, hence negating their effect. Thus, in this study, an iron-supported MOF has been synthesized for iron application. However, iron-supported MOFs are often reported to be unstable in aqueous media, which limits their application in biological and other environmental applications.^30−32^ Among them, MIL88-Fe and 101-Fe have been found to be susceptible to collapse in a polar solvent, thus showing poor hydrolytic stability.^33^ Hence, some linkers with fluorinated compounds have been used to improve the stability, which reduces the degree of biocompatibility.^34^

Hence, to form a stable Fe-MOF, here, we attempted to synthesize an amino-acid-modulated stable MOF. Here, an amino acid that has been identified to function as a glue to the leaf cuticle was used as the modulator with the aim of gaining complementary benefits.^35^ Hence, here, we developed a porous MOF platform with an amino-acid-functionalized surface acting as a molecular hand to hold nutrients against foliar washing in the presence of drift factors.

## Methods

### Materials

Ferric chloride was purchased from SRL. 2,6-Napthalene dicarboxylic acid was purchased from TCI Chemicals and used without further purification. DMF, DCM, and hexane were purchased from Merck Chemicals. The tissue-freezing medium was purchased from Leica Biosystem.

### Characterization

Powder X-ray diffraction (PXRD) was performed using a Bruker D8 advance diffractometer with a Cu Kα radiation source (λ = 1.54 Å) at 40 kV and 25 mA. Transmission electron microscopy (TEM) and high-resolution-TEM (HR-TEM) were performed using a JEOL JEM2100 at 200 kV. Thermogravimetric analysis (TGA) was performed under nitrogen conditions using a PerkinElmer STA 8000. Field-emission scanning electron microscopy (FE-SEM) was performed using a Thermo Scientific instrument. Fourier transform infrared (FTIR) spectroscopy was performed using a Vertex 70 Bruker. ζ-potential and DLS measurements were performed using a Malvern instrument. The contact angle was determined using an ADVANCE drop shape analyzer (KRUSS). Inductively coupled plasma mass spectrometry (ICP-MS) was performed using an Agilent 7700. The Brunauer–Emmett–Teller (BET) method was used to investigate the surface area of the prepared metal–organic framework. Similarly, Barrett–Joyner–Halenda desorption was used to calculate the pore size distribution from the N_2_ adsorption–desorption isotherm at 77 K with the help of the Autosorb-IQ2-MP apparatus (Quanta chrome, America).

### Synthesis of Fe-MOF

Briefly, 100 mg of FeCl_3_ was added to 50 mg of 2,6-naphthalene dicarboxylic acid in 15 mL of DMF and mixed by sonication. The mixture was then transferred to a hydrothermal vessel and allowed to react at 180 °C for 48 h. After cooling, the resultant reaction mixture was centrifuged at 7500 rpm for 20 min. The reactant were further washed with DMF three times.

### Synthesis of the Amino-Acid-Modulated MOF (MOF@aa)

Briefly, 100 mg of FeCl_3_ was added to 50 mg of 2,6-naphthalene dicarboxylic acid in 15 mL of DMF and mixed by sonication. Then, 300 mg of three amino acids (glutamine, histidine, and serine) were added separately, transferred to a hydrothermal vessel, and allowed to react at 180 °C for 48 h. Finally, the solutions were centrifuged at 7500 rpm for 20 min, washed with DMF three times, and labeled as MOF@Glu, MOF@His, and MOF@Ser, respectively.

### Activation of MOFs

The centrifuged MOFs were dried in a hot-air oven, immersed in 40 mL of DCM, sonicated, and replaced with fresh DCM three times, following an incubation time of 45–60 min after each addition. After the completion of three cycles of DCM treatment, the solvent was replaced with hexane for three cycles, with a volume of 40 mL each time. Finally, hexane was evaporated using a rotavapor, and the dried product was further characterized.

### Loading of FeSO_4_ in MOFs

Ferrous sulfate heptahydrate (FeSO_4_·7H_2_O) was loaded inside the pores of the MOF. Theoretically, the amount of ferrous sulfate that could be loaded into the porous matrix was calculated based on the pore volume. Then, a supersaturated solution of ferrous sulfate was prepared and loaded in 20 mg of prepared MOFs. The resultant mixture was stirred continuously for up to 56–60 h. Then, the mixture was centrifuged at 10 000 rpm and washed continuously three times with distilled water. The loading percent was quantified by ICP-MS.

### Release of FeSO_4_ in Water

The loaded MOF release pattern was studied in water. The loaded MOF placed in the water was incubated in a rotary shaker, and 10 μL samples were taken at subsequent intervals. The mixture was centrifuged, and the supernatant was then analyzed by ICP-MS for the Fe content. The experiment was performed in triplicate.

### Drift Analysis

The prepared MOFs were sprayed using a hand-held sprayer on tomato (*Solanum lycopersicum*) leaves distributed evenly on a Petri dish. The leaves were allowed to dry in shade for a uniform time. Then, ∼5 mL of water was sprayed to mimic rainwater, which was decanted following the spray. The MOFs resisting drift were quantified using ICP-MS through the iron analysis of the samples processed above. A similar process was performed for the control samples. The experiment was performed in triplicate.

### Nutrient Delivery Efficiency

Two-week-old tomato plants were uprooted after sowing and transferred into iron-free Johnson’s hydroponic growth solution. The plants were grown in an iron-free medium for up to another 2 weeks until chlorosis appeared. Then, the loaded iron (MOFs) were prepared in water and spotted onto the leaves using a capillary tube and samples were taken at intervals of 12 and 24 h to visualize the cross sections of the leaf by FE-SEM and analyze the Fe content through EDAX measurement.

### Cross Sections of the Leaves

Leaves were excised from the control and treated tomato plants. The collected sample was first washed in distilled water, followed by submerging the samples in the fixing solution containing 4% paraformaldehyde and 0.1% glutaraldehyde, followed by incubation overnight at 4 °C. After incubation, samples were washed twice in phosphate-buffered saline (PBS, pH 7.4) for 5 min each. The leaves were then subjected to a 2.3 M sucrose gradient (25, 33, 50, 66, 75, and 100% sucrose) infiltration. The infiltration steps include the incubation of leaf samples in a gradient of 25, 33, 50, 66, and 75% for 1 h at room temperature, followed by overnight incubation with 100% 2.3 M sucrose at 4 °C.

### Cryosectioning

Leaf tissues (approximately 1 cm^2^) were embedded into the tissue-freezing medium and frozen at −30 °C in a Leica CM 1950 Cryostat. The frozen blocks with samples were trimmed, and 30–50 μm thick samples were sectioned until the region of interest was obtained. The section was then carefully placed on a positively charged glass slide and stored at 4 °C until imaging. Frozen blocks containing the embedded tissue were stored at −80 °C.

## Results and Discussion

### Material Characterization

In this study, foliar application of micronutrients to the plant was planned using Fe-MOF and Fe-MOF@aa (amino acids) to conclude whether the aa can modulate the size/shape, as well as to aid in the binding to the leaf surface against the drift. The Fe-MOF was synthesized using an Fe metal node, 2,6-naphthalene dicarboxylic acid (NDC) as an organic linker, and an amino acid modulator (MOF@aa) (viz., serine, histidine, and glutamine). The hydrothermal method was employed, and the products were denoted as MOF@Ser, MOF@His, and MOF@Glu, respectively.

**Figure 1:**
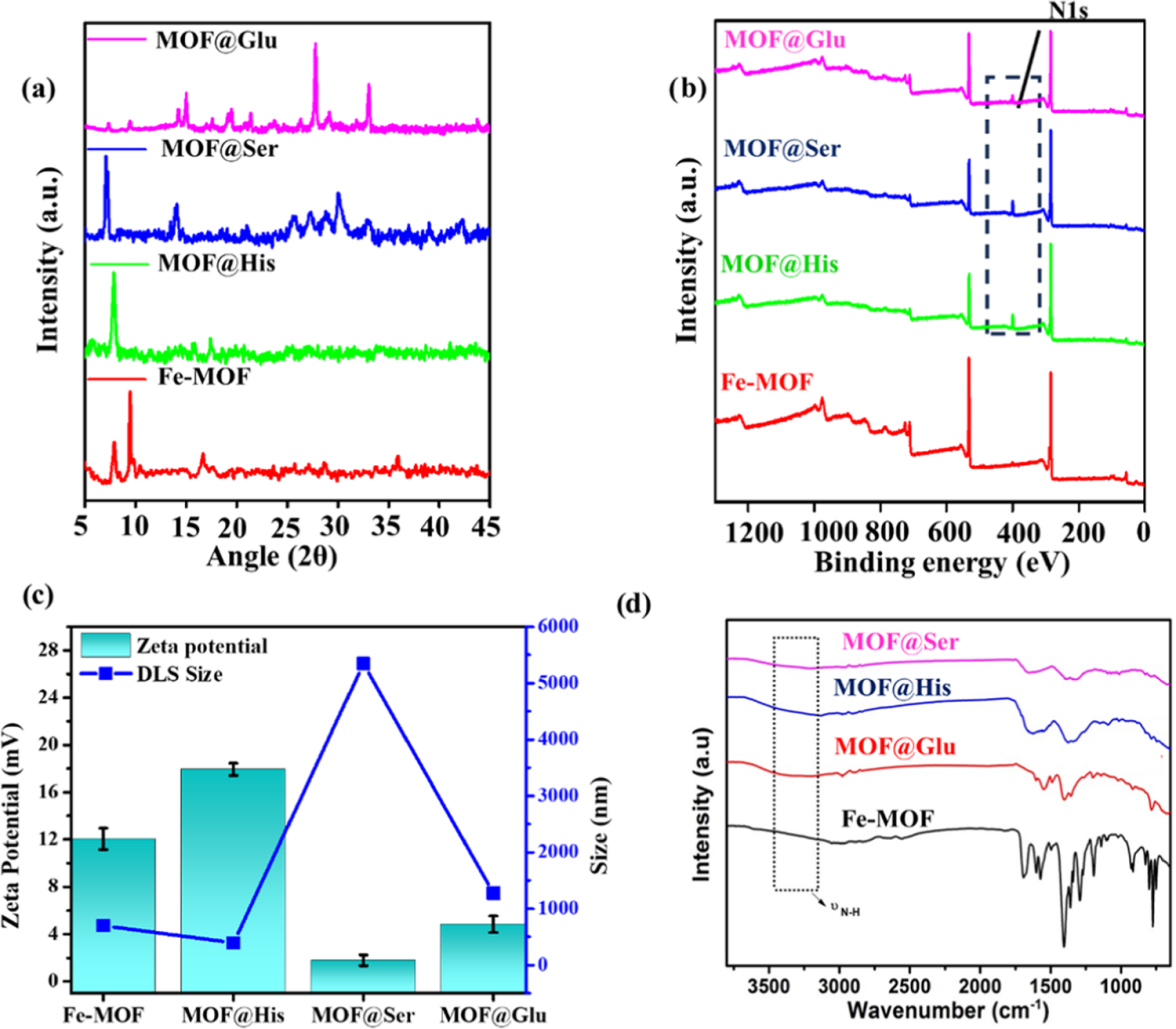
Characterization of MOFs: (a) PXRD of Fe-MOF and MOF@aa, i.e., MOF@His, MOF@Ser, and MOF@Glu. (b) XPS spectra of Fe-MOF and MOF@aa. (c) ζ-potential expressed in mV and DLS size for the prepared Fe-MOF and MOF@aa. (d) FTIR for Fe-MOF and MOF@aa.

The XRD analysis of the Fe-MOF samples without amino acids shows a major peak at 9 and 16.7°, which corresponds to the (010) and (110) planes of the 3-dimensionally grown NDC-linked MOF, respectively (Figure 1a).^36,37^ MOF@aa significantly differs in the diffraction pattern; this may be because of the change in the binding preference of each amino acid to different growing organic pillars of the MOF or the susceptibility of the long-chain linker to interpenetration. The XRD peaks of MOF@Ser at 7.1, 14.2, and 21° correspond to the peak reported from SUMOF3, with a similar linker and Fe/Zn nodes.^38,39^ However, MOF@His shows two peaks (7.9 and 17.6°), which indicates the growth of the MOF to be limited in one direction to a 2D structure. Finally, in MOF@Glu, multiple peaks were evidenced, which may be from the interpenetrated structure of the MOF. The amino acid concentration was optimized such that it is at a higher ratio relative to other precursor salts so that the above stable XRD pattern is obtained.

X-ray photoelectron spectroscopy (XPS) was conducted on pristine MOF and amino-acid-functionalized MOF samples in order to investigate the bonding between the MOF and amino acids. XPS confirms the presence of Fe, C, and O elements in pristine MOF and additional N in all amino-acid-functionalized MOFs (as shown in Figure 1b), which also validates the fact that serine, histidine, and glutamine have been bonded to pristine MOF.

The oxidation state of Fe^3+^ in Fe 2p is related to the peak positions of about 711.1 eV (Fe 2p_3/2_) and 724.4 eV (Fe 2p_1/2_). The three peak points in the C 1s spectrum are at 284.3, 284.8, and 288.3 eV, which are ascribed to the C=C in the naphthalene ring of NDC, the C–O, and the O–C=O, respectively. In the O 1s spectrum, the absorbed water molecule peak O–H is at 532.5 eV; the peak at 532 eV corresponds to COOH binding of NDC; and the Fe–OH and Fe–O peaks are present at 531.2 and 529.7 eV, respectively, as shown in Figure S1.^40^

The presence of the N 1s peak in all amino-acid-functionalized MOF samples marks the presence of amino acids. In serine and glutamine MOF samples, the high-resolution N 1s peaks at 398.6 and 400 eV are attributed to pyridinic and quaternary nitrogen species, respectively, as shown in Figure S2.^41^ However, in the histidine MOF N 1s spectra, peaks at 398.5, 399.9, and 400.7 eV are attributed to the pyridinic, pyrrolinic, and quaternary nitrogen species, respectively (Figure S3).^42^

#### ζ-Potential and DLS Size Measurement

The zeta potential and DLS of the prepared MOFs were assessed in water, which indicates their solubility (Figure 1c). The DLS size order corroborates the TEM image of Fe-MOF in comparison to MOF@aa. Also, it was observed that the DLS size of MOF@Ser was the highest, which was the largest in the TEM image also, possibly due to the instability of the colloidal suspension during the measurements. The instability may be due to the least surface charge, which corroborates the ζ-potential measurement. MOF@Glu shows a larger size than MOF@His, possibly due to the formation of cluster association as visualized in the SEM and TEM images. Among the samples, MOF@His was stable; hence, the TEM image shows the least size, which may be due to its high surface charge.

Further, to confirm the amino-acid-functionalized Fe-MOF formation, FTIR analysis was performed. As shown in Figure 1d, all of the IR bands correspond well with the existing Fe-MOF literature.^40^ The area of the spectrum between 1150 and 1700 cm^–1^ represents the distinctive peaks of the carboxylate groups of NDC. The peaks found at 1566 and 1404 cm^–1^ correspond to asymmetric and symmetric carboxyl group vibrations, respectively. The vibration of the C–H bond in the benzene ring is responsible for the adsorption peak at 772 cm^–1^. The vibrations below 600 cm^–1^ correspond to metal linkers. These vibrational modes are difficult to identify using the IR technique. FTIR spectra of MOF@aa exhibit its parent MOF characteristic peaks as well as an additional broad peak around 3200 cm^–1^ attributed to stretching vibrational modes from the N–H group.^43^ This demonstrates that the MOF has been effectively functionalized with amino acids, i.e., MOF@aa (Figure 1d). Also, the UV–vis absorbance spectra of the prepared MOFs are displayed in the Supporting Information (Figure S4).

**Figure 2:**
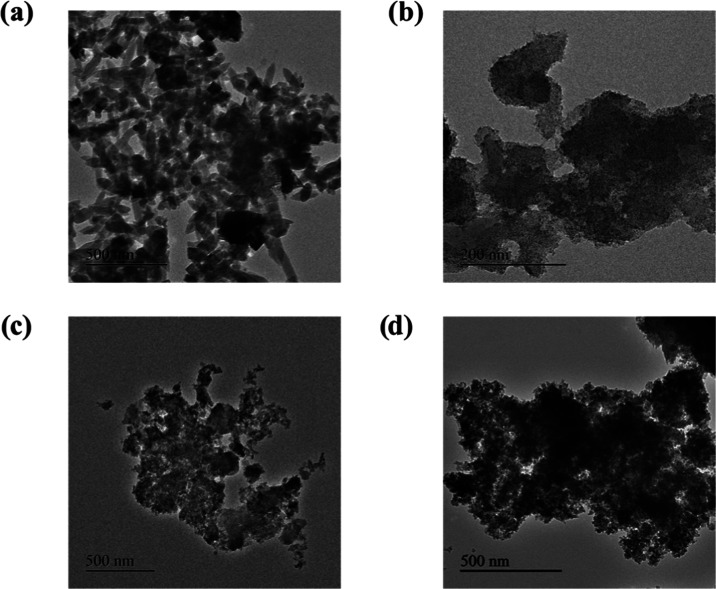
TEM images of different MOFs: (a) Fe-MOF, (b) MOF@His, (c) MOF@Glu, and (d) MOF@Ser.

The TEM image of the Fe-MOF sample shows a rod-shaped rigid structure, which is typical of a 2,6-NDC MOF (Figure 2a). This may be due to the rapid growth of the less nucleated metal and carboxylate structure,^44^ whereas, in the samples prepared with the amino acids, the termination of the growth by the modulator limited the particle size to smaller than that of Fe-MOF. Among the MOF@aa samples, the size distribution is in the following order: viz., MOF@Glu < MOF@His < MOF@Ser (Figure 2b–d). The quick nucleation and growth termination cause the structure to not look like a rod. Corresponding SEM images of the samples are provided in the Supporting Information (Figure S5–S8).

**Figure 3:**
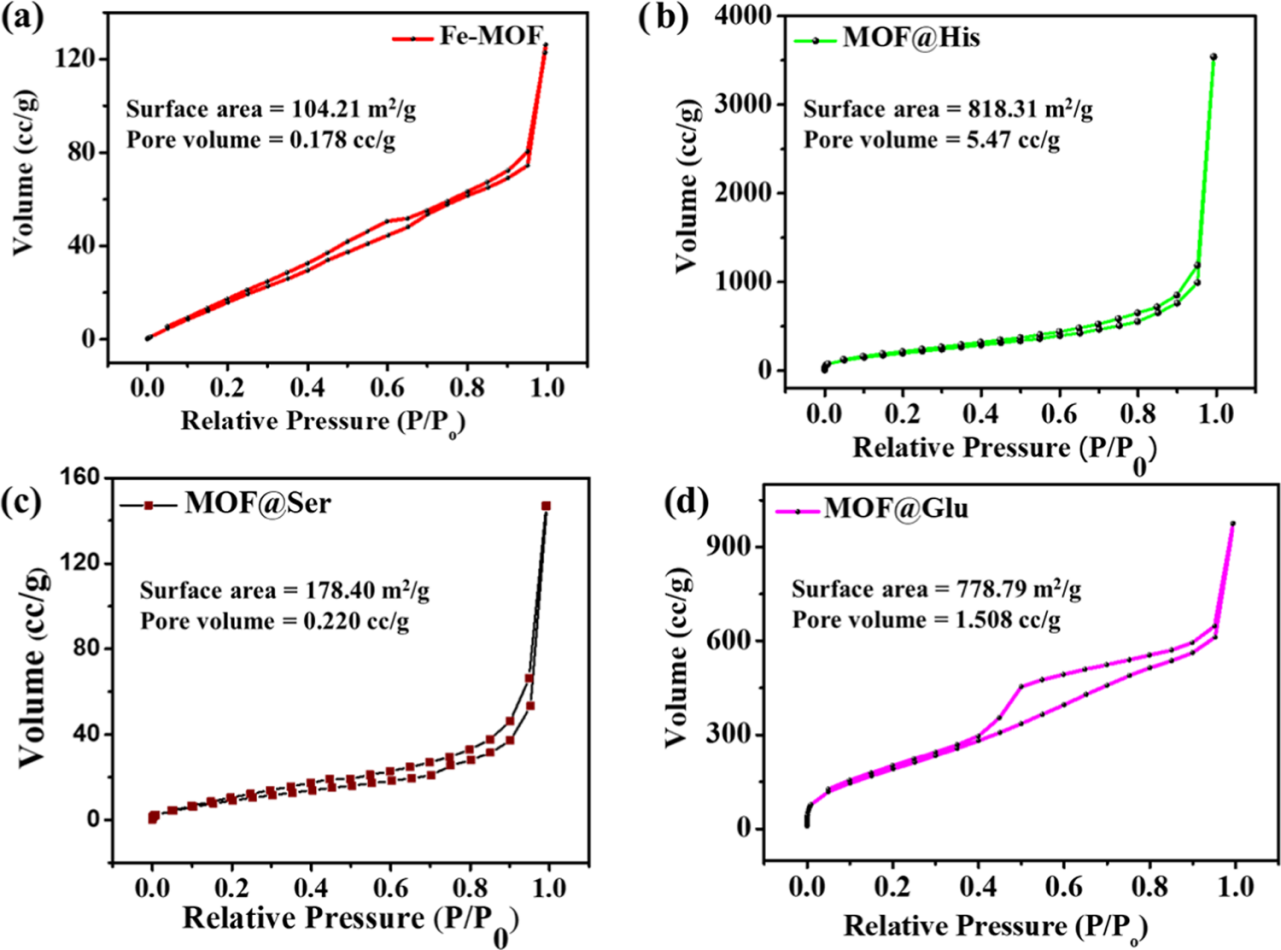
BET surface area of different MOFs: (a) Fe-MOF, (b) MOF@His, (c) MOF@Ser, and (d) MOF@Glu.

Further, the surface area measurement of the different MOF samples by the BET analyzer shows Fe-MOF and MOF@Ser to have a type 3 isotherm pattern (Figure 3a,c). However, the MOFs prepared using MOF@Glu and MOF@His show a type 2 isotherm pattern (Figure 3b,d). Fe-MOF and MOF@Ser samples show a slight increase in the surface area up to about 1 *P*/*P*_o_ partial pressure, whereas MOF@Glu and MOF@His show an upward trend in the surface area, which reaches up to about ≥75 cc g^–1^ under 0.01 *P*/*P*_o_ partial pressure.^45^ This shows the ability of amino acids, i.e., glutamine and histidine, to modulate the surface area of the samples to ∼800 m^2^ g^–1^ by allowing the loss of free solvent during the activation (Figure 3b,d).^9^ The control and serine MOFs may not allow the solvent to completely leave during activation, which causes the surface area to be <200 m^2^ g^–1^. The reduced surface area could be due to its inherent nature to aggregate, which reduces its surface area significantly. The bimodal pore distribution may be due to the defects in the internal MOF structure.^46,47^ The predicted structure of the MOF is given in the Supporting Information (Figure S9).

### Loading and Release

**Figure 4:**
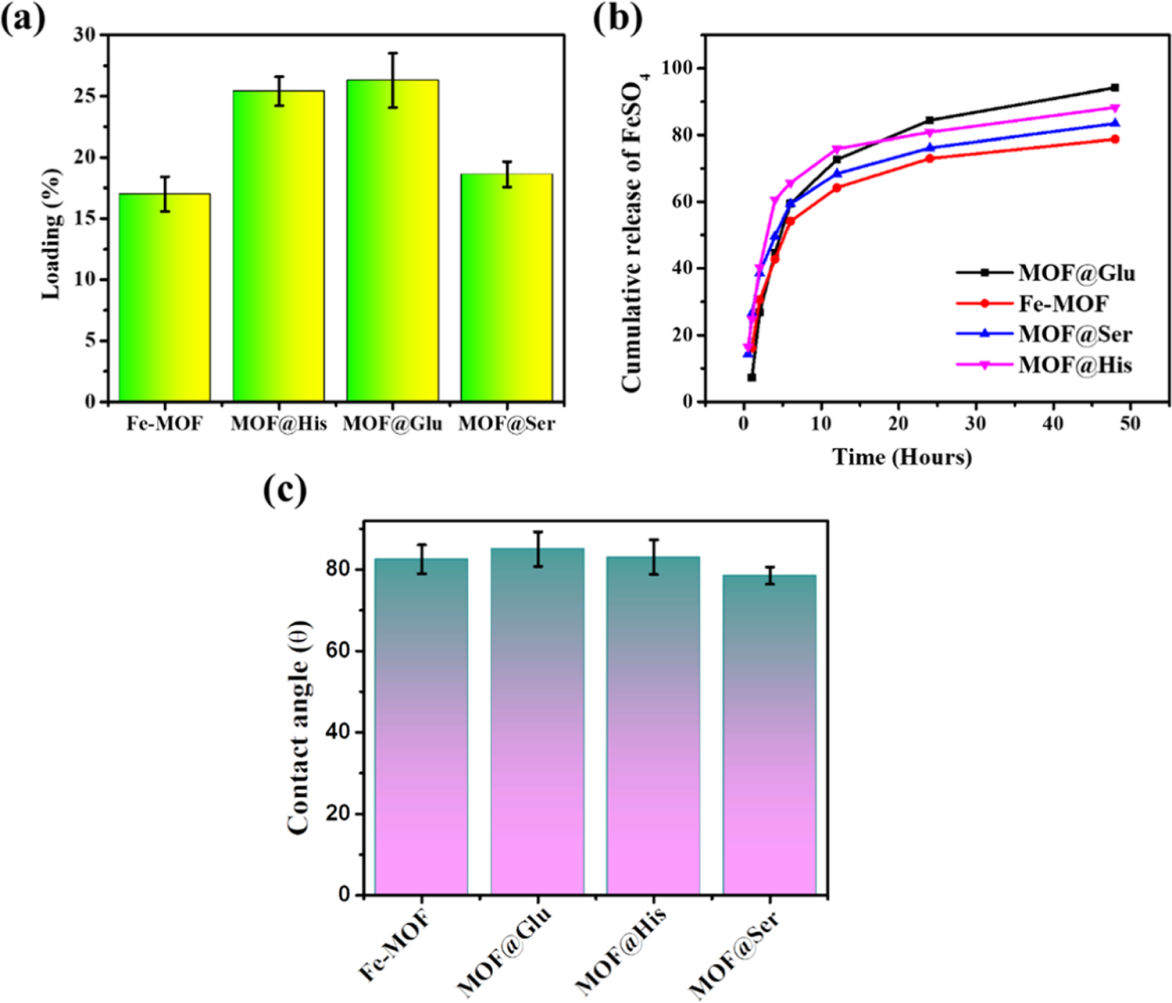
(a) Loading profiles of the prepared Fe-MOF and MOF@aa for the iron micronutrient. (b) Time-dependent iron release at room temperature for the loaded MOFs quantified using ICP-MS. (c) Contact angle of the prepared Fe-MOF and MOF@aa.

The promising role of MOFs in the controlled release has to be investigated before proceeding to the plant experiment. For this investigation, FeSO_4_-loaded Fe-MOF/Fe-MOF@aa was incubated in water to collect the samples at desired intervals and estimate the iron content through ICP-MS. (Figure 4a). The loadings in MOF@Glu and MOF@His are higher due to their larger pore volumes compared to other MOFs. In this analysis, Fe-MOF shows the least release, which is obviously due to the less pore surface and smaller pore size. MOF@Ser shows marginally more release compared with Fe-MOF.

However, MOF@Glu and MOF@His show the maximum release among all samples. In comparison to MOF@His and MOF@Glu, MOF@His shows the maximum release in the initial period, followed by a slower release at 24 h, which may be due to bimodal pore distribution or bimodal binding. This leads to an initial noncapillary-bound nutrient release, followed by a later capillary-bound nutrient release (Figure 4b). However, MOF@Glu shows a slower release in the initial observation, which significantly ramped after 12 h and surpassed the release speed of MOF@His in 24 h. This may be because of a pore architecture wherein the initial nutrient blocks the pore, whose release paves the way for the remaining interior nutrient to flow out quickly.

#### Contact Angle

The contact angle for the prepared MOFs was assessed by using a compressed pellet sample. All of the prepared MOFs show a contact angle less than 90°, imparting hydrophilicity to the prepared MOF structure. This degree of hydrophilicity is suitable for foliar application (Figure 4c).

### Drift Analysis

**Figure 5:**
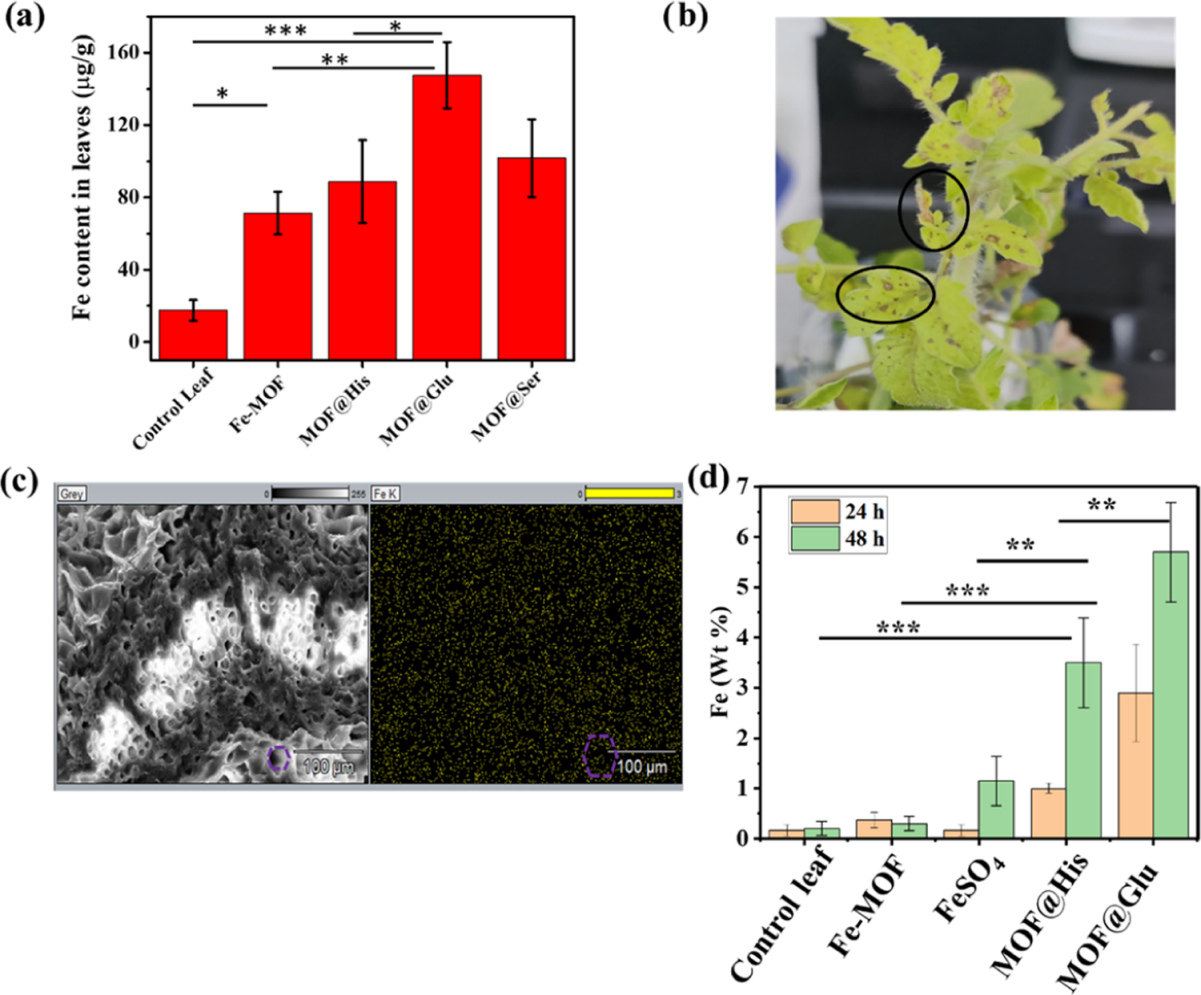
(a) Effect of simulated water drift on the ability of different MOFs to be retained on the leaf surface. (b) Toxicity effect of MOF@Glu in tomato leaves. (c) FE-SEM image and Fe elemental mapping on the cross sections of the leaf, showing the colocalization of the element on the leaf structure, with similar pore gaps. (d) Fe wt % obtained from the EDAX analysis on cross sections of the leaf samples treated with different iron-loaded MOF samples.

The aim of this study is to examine the effect of amino acids on the MOF to hold on to the cuticle and avoid nutrient loss. In order to quantify this, MOF samples without amino acids and with amino acids were sprayed on the model plant, viz., tomato, and challenged against simulated rain. It is clear from the study that MOF@Glu was able to hold to the cuticle strongly, followed by MOF@His. The other MOF samples, i.e., MOF@Ser and Fe-MOF, displayed the least cuticle-holding capacity (Figure 5a). In addition to the increased binding capacity of glutamine and histidine to overcome the drift, the structure of the particles may also have played a role. Fe-MOF shows a rod shape, and MOF@Ser shows a spherical morphology, which is prone to roll compared to the 2D structures of MOF@Glu and MOF@His. As the porosity, loading, and resistance to drift are poor in both Fe-MOF and MOF@Ser samples, further plant experiments were limited to studying the efficiencies of MOF@His and MOF@Glu in comparison to Fe-MOF.

As foliar application has often been noticed to cause toxicity, it is pertinent to test the influence of MOFs on foliar toxicity. For this test, the different MOF samples were spotted on the leaf using a capillary tube, following which the burning spots appearing were enumerated. In this experiment, the tomato plant that received glutamine MOF was noticed to exhibit a significant number of leaf burn spots (Figure 5b,c), whereas in the plant that received Fe-MOF as well as MOF@His, the spots were much fewer in number. The relatively higher foliar toxicity of MOF@Glu may be due to the higher metal content in the sample, which was inferred from the TGA analysis (Figure S10). The MOF@His and MOF@Glu samples investigated using TGA show a typical sharp reduction in the weight of the MOF up to 100 °C in comparison to the control, which corresponds to physically attached water,^48^ followed by a gradual reduction up to 250 °C corresponding to the chemically attached water. This gradual reduction extended up to 400 °C in the control, which may be due to the smaller pore size, which does not allow water to leave the pore as quickly as in the other two samples, where the pore size is larger. Finally, the metal content of MOF@Glu samples was found to be marginally higher than those of the other samples, which is also reflected in the loaded sample quantification.

### Nutrient Delivery Efficiency

Finally, to compare the effects of different MOFs on the foliar nutrient application, the MOF loaded with FeSO_4_ was sprayed on the tomato plant, and the cross section of the leaf tissue was evaluated after a 24 h time interval. In this evaluation, the plant that received the foliar nutrient through MOF@His was found to show the highest iron content in the leaf (Figure 5d), whereas Fe-MOF and MOF@Glu showed poor nutrient delivery efficiency. This may be because Fe-MOF does not hold the required amount of iron and MOF@Glu causes burn spots, which do not allow the nutrient to reach the live tissue.

## Conclusions

In this work, amino-acid-modulated MOFs were synthesized for the loading and sustainable release of iron to crops through the foliar route. Among the amino acids, serine, histidine, and glutamine were used as modulators as they are reported to show relatively stronger binding to the leaf cuticle. Thus, the synthesized MOFs, viz., MOF@Ser, MOF@His, and MOF@Glu, reduced the size of the MOF in the order glutamine < histidine < serine. MOF@His and MOF@Glu showed a higher surface area, whereas Fe-MOF and MOF@Ser showed a lower surface area. Hence, the plant experiment was limited to MOF@His and MOF@Glu in comparison with Fe-MOF. In this analysis, MOF@Glu shows higher foliar toxicity and poor iron supply to the plant, whereas MOF@His showed lower toxicity and effective supply of the nutrient to the leaf. The cost of this technique raises a reasonable doubt, but this depends on the crop, and also, future low-cost techniques can make this a practical technique.

## Supporting Information Available

XPS spectra (Figure S1–S3); UV–vis absorbance (Figure S4); SEM images (Figure S5–S8); predicted structure of the MoF (Figure S9); and TGA curves (Figure S10) (PDF)
