# Supplementary material for: Bells and Whistles on Fertilizers: Molecular Hands to Hang Nanoporous Foliar Fertilizer Reservoirs
Source: ACS Omega. 2024 Jun 5;9(24):25870–8. doi: 10.1021/acsomega.3c09895 (PMC11191114; doi:10.1021/acsomega.3c09895)
Supplement: Supplementary file 1 — ao3c09895_si_001.pdf [file ao3c09895_si_001.pdf]

Bells and whistles on fertilizer: molecular hands to hang nano-porous foliar fertilizer reservoir

Kamaljit Kaur,[1] Mahima Chandel,[2] Poonam Sagar,[3] Bandana Kumari Sahu,[2] Ritu Ladhi, [2] Parameswaran Rajamanickam,[2] Pooja Aich,[2] Madhu Khatri,[1] Selvaraju Kanagarajan,[3]* Nitin Kumar Singhal,[3]* Monika Singh,[2]* and Vijaya Kumar Shanmugam[2]*

[1]University Institute of Engineering and Technology, Panjab University, Chandigarh

[2] Institute of Nano Science and Technology, Sector- 81, S.A.S. Nagar, Mohali, Punjab – 140306, India

[3] Department of Plant Breeding, Swedish University of Agricultural Sciences, 234 22 Lomma, Sweden

[4] Food and Nutritional Biotechnology, National Agri-Food Biotechnology Institute, Mohali, Punjab, India

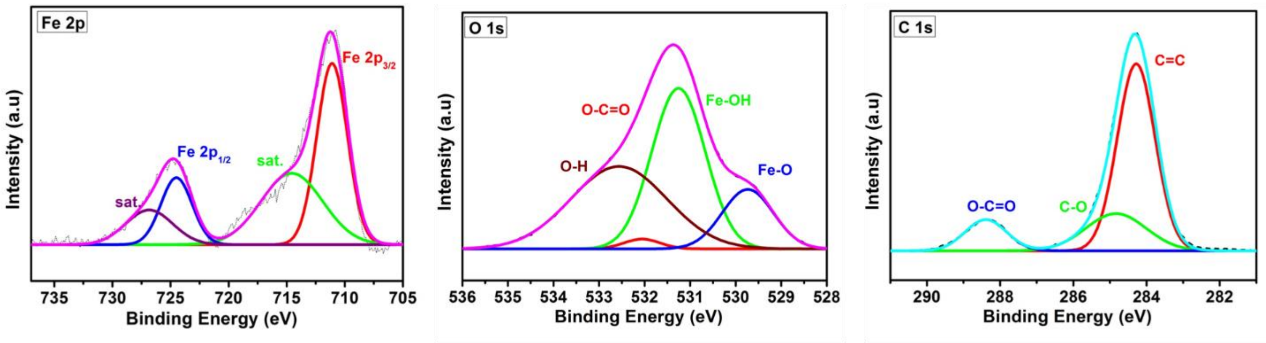

Figure S1: XPS spectra of the prepared Fe-MOF

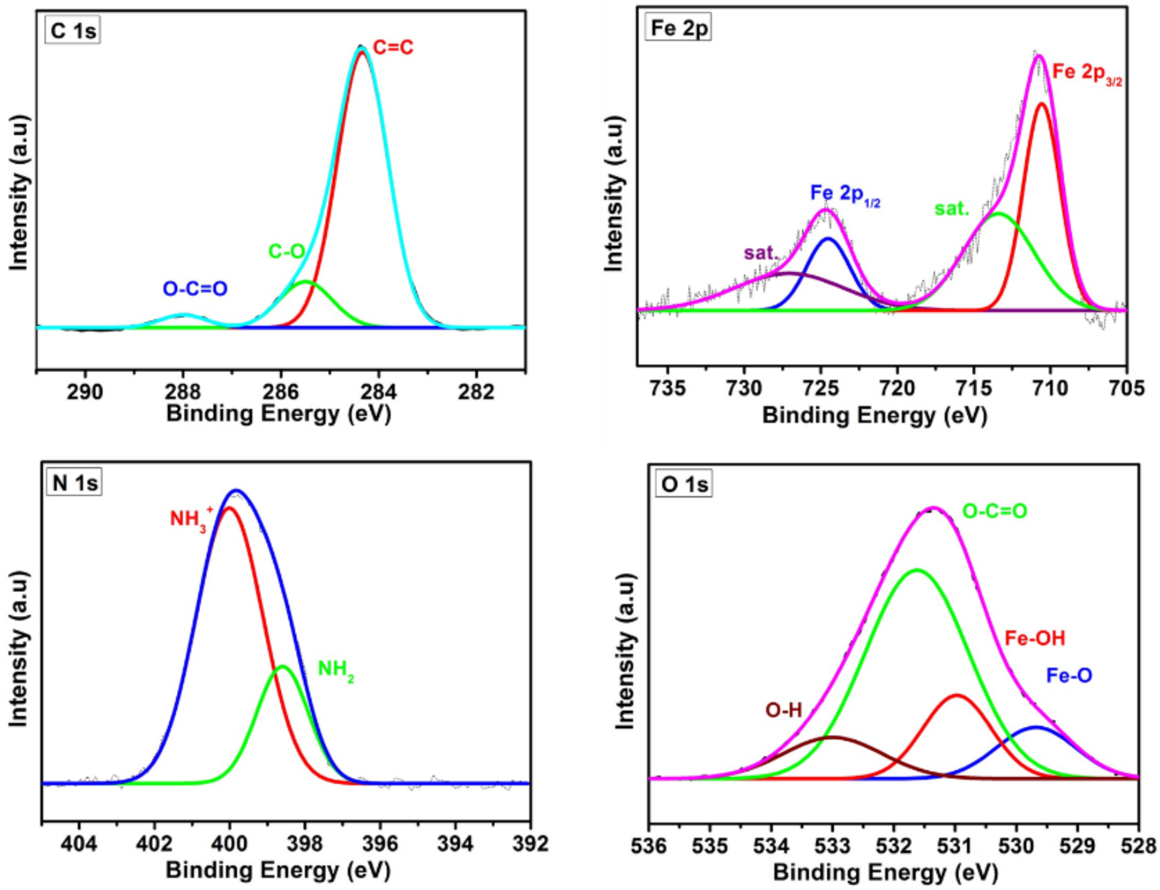

Figure S2: XPS spectra of MOF@Glu

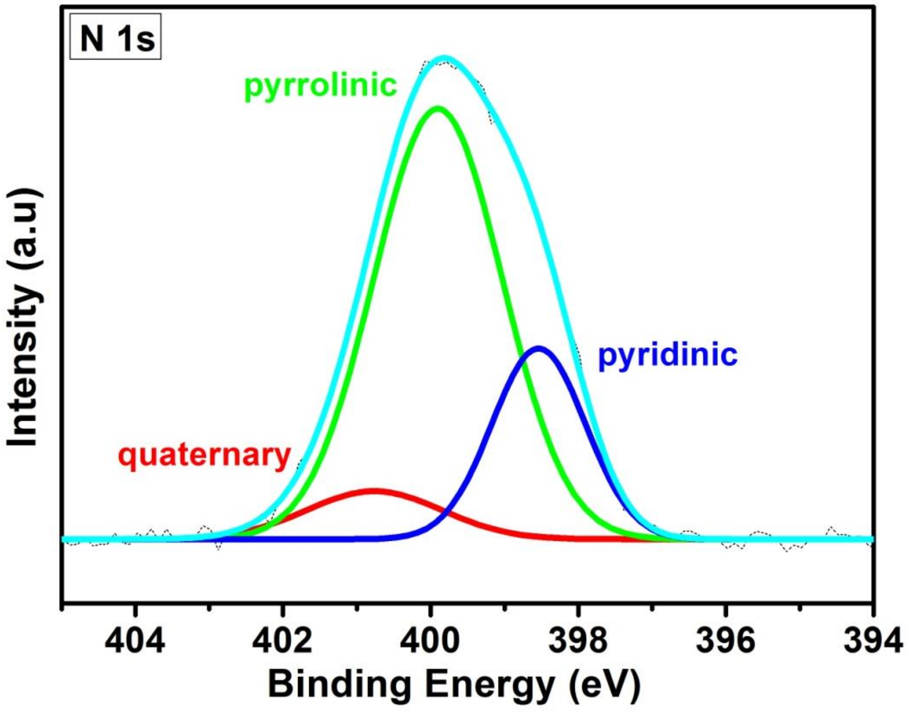

Figure S3: XPS spectra of MOF@His

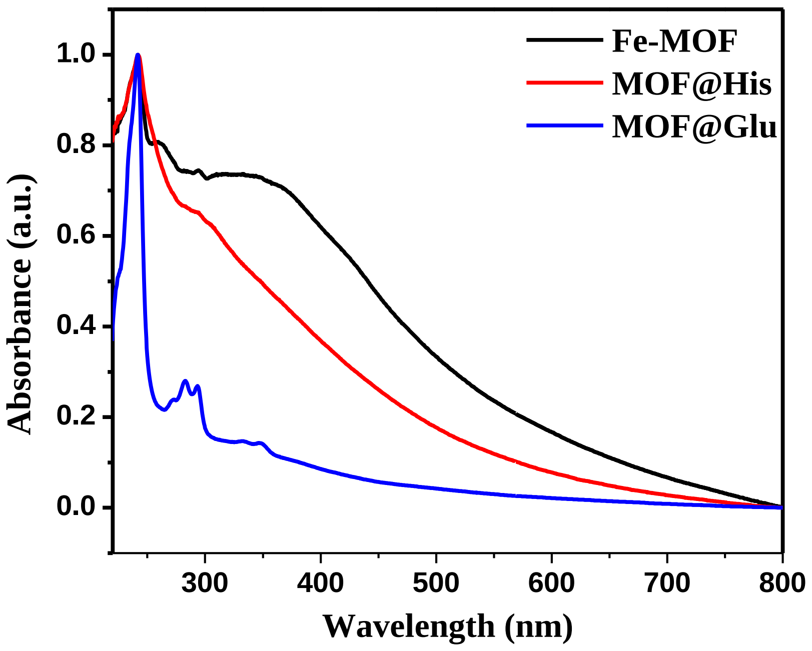

Figure S4: UV-Vis absorbance spectra of prepared MOFs with the amino acid shows wide absorbance which suits to be active ingredient carrier.

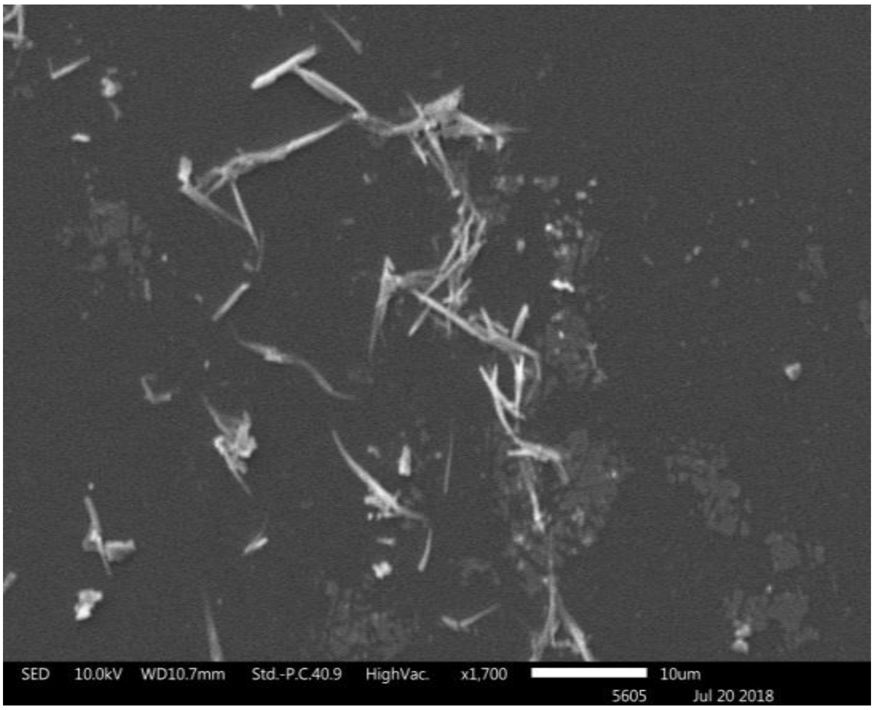

Figure S5: SEM image of FE-MOF

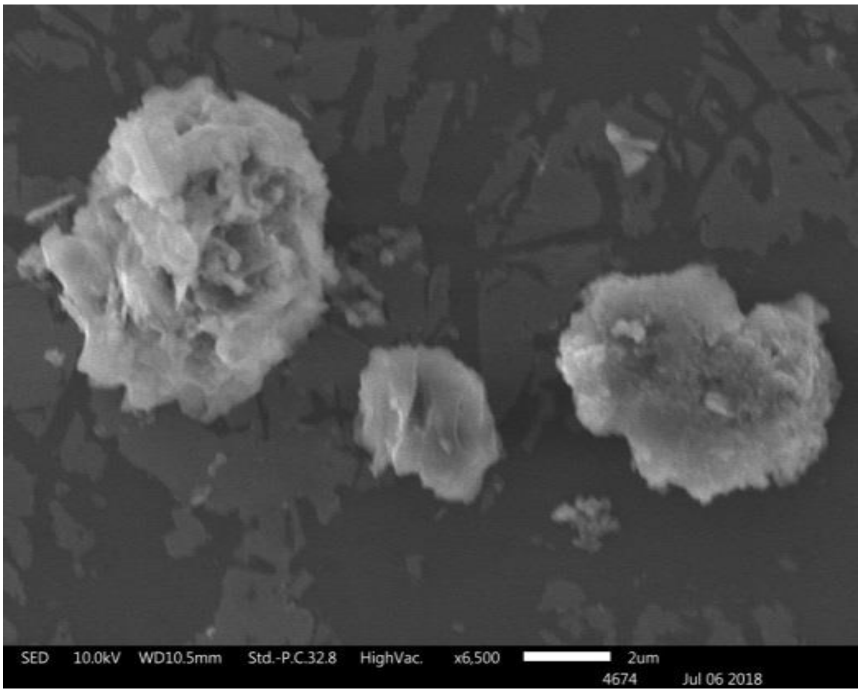

Figure S6: SEM image of MOF@Ser

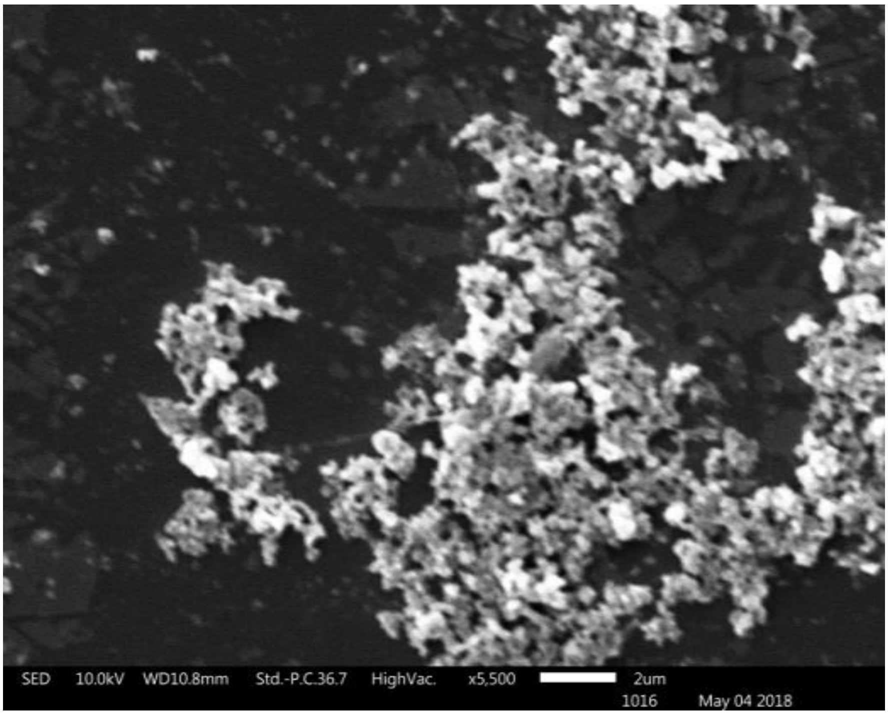

Figure S7: SEM image of MOF@His

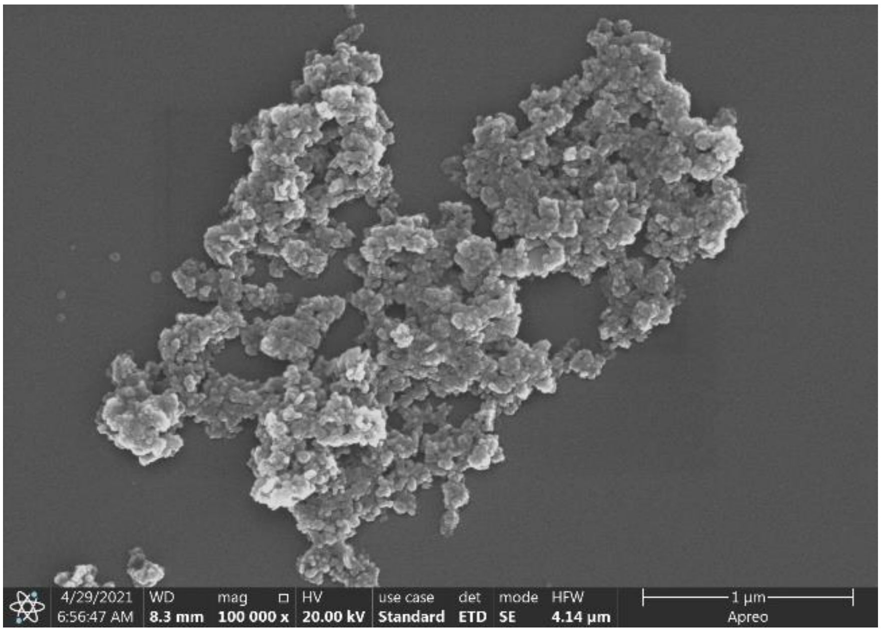

Figure S8: SEM image of MOF@Glu

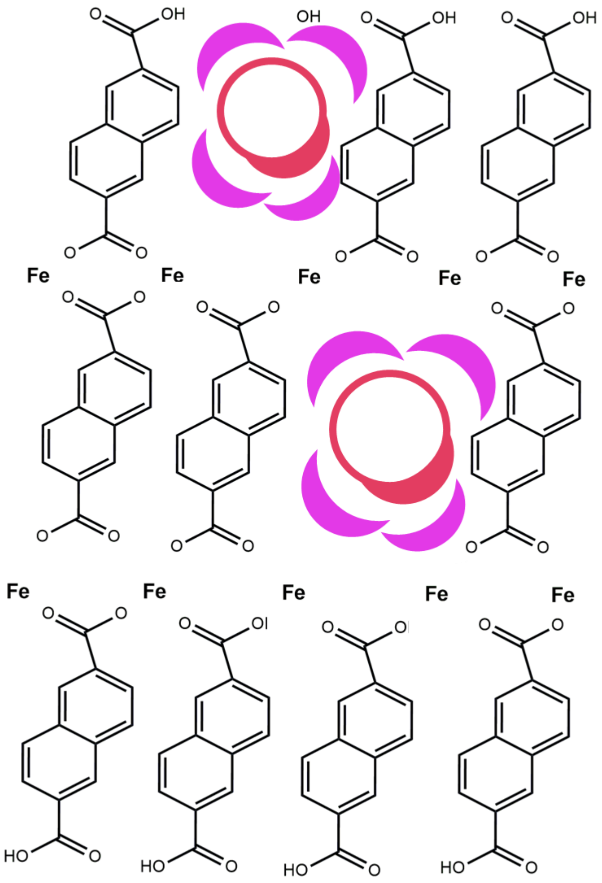

Figure S9: predicted structure of the MOF (pink crescent shows the blocking of the metal growth centre by the amino acid and assembly of the same)

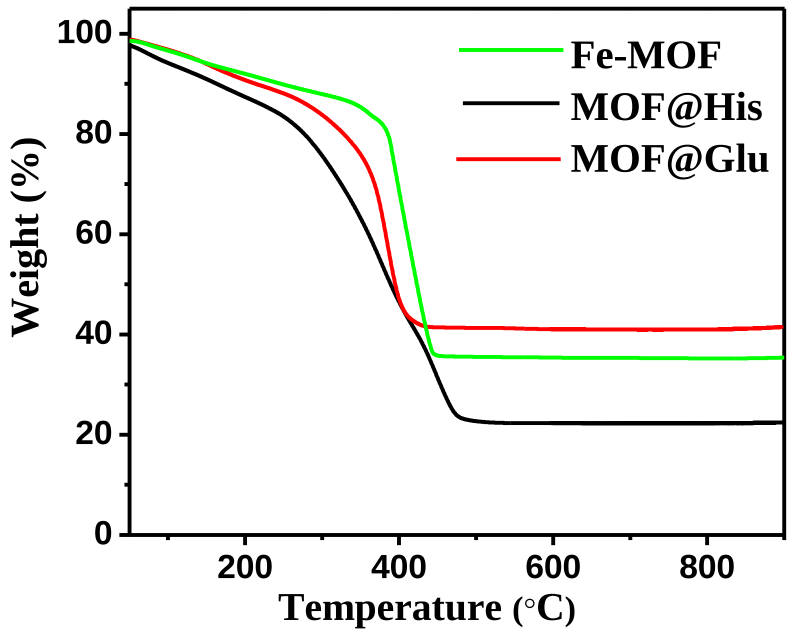

Figure S10: Thermogravimetric analysis (TGA) of Fe-MOF, MOF@His and MOF@Glu
